# Supplementary material for: Resolving the full spectrum of human genome variation using Linked-Reads
Source: Genome Res. 2019 Apr;29(4):635–45. doi: 10.1101/gr.234443.118 (PMC6442396; doi:10.1101/gr.234443.118)
Supplement: Supplemental Material [file supp_gr.234443.118_Supplemental_longranger-2.2.zip › longranger-2.2.tar.bz2!/lr2.2-public/lib/stan/cmdstan/stan/doc/api/html/valued__argument_8hpp_source.html]

Stan: src/stan/gm/arguments/valued\_argument.hpp Source File


|  |  |
| --- | --- |
|  | Stan  2.5.0  probability, sampling & optimization |


- Main Page
- Related Pages
- Namespaces
- Classes
- Files

- File List
- File Members

All Classes Namespaces Files Functions Variables Typedefs Enumerations Enumerator Friends Macros Pages

- src
- stan
- gm
- arguments

valued\_argument.hpp

Go to the documentation of this file.

1 #ifndef STAN\_\_GM\_\_ARGUMENTS\_\_VALUED\_\_ARGUMENT\_\_BETA

2 #define STAN\_\_GM\_\_ARGUMENTS\_\_VALUED\_\_ARGUMENT\_\_BETA

3

4 #include <stan/gm/arguments/argument.hpp>

5

6 namespace stan {

7

8  namespace gm {

9

10  class valued\_argument: public argument {

11

12  public:

13

14  virtual void print(std::ostream\* s, const int depth, const std::string prefix) {

15  if (!s)

16  return;

17

18  std::string indent(compute\_indent(depth), ' ');

19

20  \*s << prefix << indent << \_name << " = " << print\_value();

21  if(is\_default())

22  \*s << " (Default)";

23  \*s << std::endl;

24  }

25

26  virtual void print\_help(std::ostream\* s, const int depth, const bool recurse = false) {

27  if (!s)

28  return;

29

30  std::string indent(indent\_width \* depth, ' ');

31  std::string subindent(indent\_width, ' ');

32

33  \*s << indent << \_name << "=<" << \_value\_type << ">" << std::endl;

34  \*s << indent << subindent << \_description << std::endl;

35  \*s << indent << subindent << "Valid values:" << print\_valid() << std::endl;

36  \*s << indent << subindent << "Defaults to " << \_default << std::endl;

37  \*s << std::endl;

38

39  }

40

41  virtual std::string print\_value() = 0;

42  virtual std::string print\_valid() = 0;

43  virtual bool is\_default() = 0;

44

45  protected:

46

47  std::string \_default;

48  std::string \_value\_type;

49

50  };

51

52  } // gm

53 } // stan

54 #endif

stan::gm::valued\_argument::print\_help

virtual void print\_help(std::ostream \*s, const int depth, const bool recurse=false)

**Definition:** valued\_argument.hpp:26

stan::gm::valued\_argument::\_default

std::string \_default

**Definition:** valued\_argument.hpp:47

argument.hpp

stan::gm::valued\_argument

**Definition:** valued\_argument.hpp:10

stan::gm::valued\_argument::print\_valid

virtual std::string print\_valid()=0

stan::gm::argument::compute\_indent

int compute\_indent(const int depth)

**Definition:** argument.hpp:71

stan::gm::valued\_argument::is\_default

virtual bool is\_default()=0

stan::gm::valued\_argument::\_value\_type

std::string \_value\_type

**Definition:** valued\_argument.hpp:48

stan::gm::argument::\_name

std::string \_name

**Definition:** argument.hpp:76

stan::gm::argument::indent\_width

int indent\_width

**Definition:** argument.hpp:79

stan::gm::valued\_argument::print

virtual void print(std::ostream \*s, const int depth, const std::string prefix)

**Definition:** valued\_argument.hpp:14

stan::gm::valued\_argument::print\_value

virtual std::string print\_value()=0

stan::gm::argument

**Definition:** argument.hpp:12

stan::gm::argument::\_description

std::string \_description

**Definition:** argument.hpp:77


---

[ Stan Home Page ]

*© 2011–2014,
Stan Development Team.*
